# Harnessing exosomes in theranostic applications: advancements and insights in gastrointestinal cancer research

**DOI:** 10.1007/s12672-024-01024-x

**Published:** 2024-05-14

**Authors:** Ali Shojaeian, S. R. Naeimi Torshizi, Mahsa Sadat Parsapasand, Zahra Sobhi Amjad, Ali Khezrian, Abbas Alibakhshi, Faye Yun, Kaveh Baghaei, Razieh Amini, Stevan Pecic

**Affiliations:** 1 Research Center for Molecular Medicine, Hamadan University of Medical Sciences, Hamadan, Iran; 2 Department of Microbiology, School of Medicine, Kermanshah University of Medical Sciences, Kermanshah, Iran; 3 Department of Chemistry and Biochemistry, California State University, Fullerton, USA; 4 Olivia Newton-John Cancer and Research Institute, Melbourne, VIC Australia; 5 School of Cancer Medicine, La Trobe University, Melbourne, VIC Australia; 6 Basic and Molecular Epidemiology of Gastrointestinal Disorders Research Center, Research Institute for Gastroenterology and Liver Diseases, Shahid Beheshti University of Medical Sciences, Tehran, Iran

**Keywords:** Exosomes, Gastrointestinal cancer, Cancer therapy, Biomarker

## Abstract

Exosomes are small extracellular vesicles (30–150 nm) that are formed by endocytosis containing complex RNA as well as protein structures and are vital in intercellular communication and can be used in gene therapy and drug delivery. According to the cell sources of origin and the environmental conditions they are exposed to, these nanovesicles are very heterogeneous and dynamic in terms of content (cargo), size and membrane composition. Exosomes are released under physiological and pathological conditions and influence the pathogenesis of cancers through various mechanisms, including angiogenesis, metastasis, immune dysregulation, drug resistance, and tumor growth/development. Gastrointestinal cancer is one of the deadliest types of cancer in humans and can involve organs e.g., the esophagus and stomach, or others such as the liver, pancreas, small intestine, and colon. Early diagnosis is very important in this field because the overall survival of patients is low due to diagnosis in late stages and recurrence. Also, various therapeutic strategies have failed and there is an unmet need for the new therapeutic agents. Exosomes can become promising candidates in gastrointestinal cancers as biomarkers and therapeutic agents due to their lower immunity and passing the main physiological barriers. In this work, we provide a general overview of exosomes, their biogenesis and biological functions. In addition, we discuss the potential of exosomes to serve as biomarkers, agents in cancer treatment, drug delivery systems, and effective vaccines in immunotherapy, with an emphasis on gastrointestinal cancers.

**Graphical Abstract:**

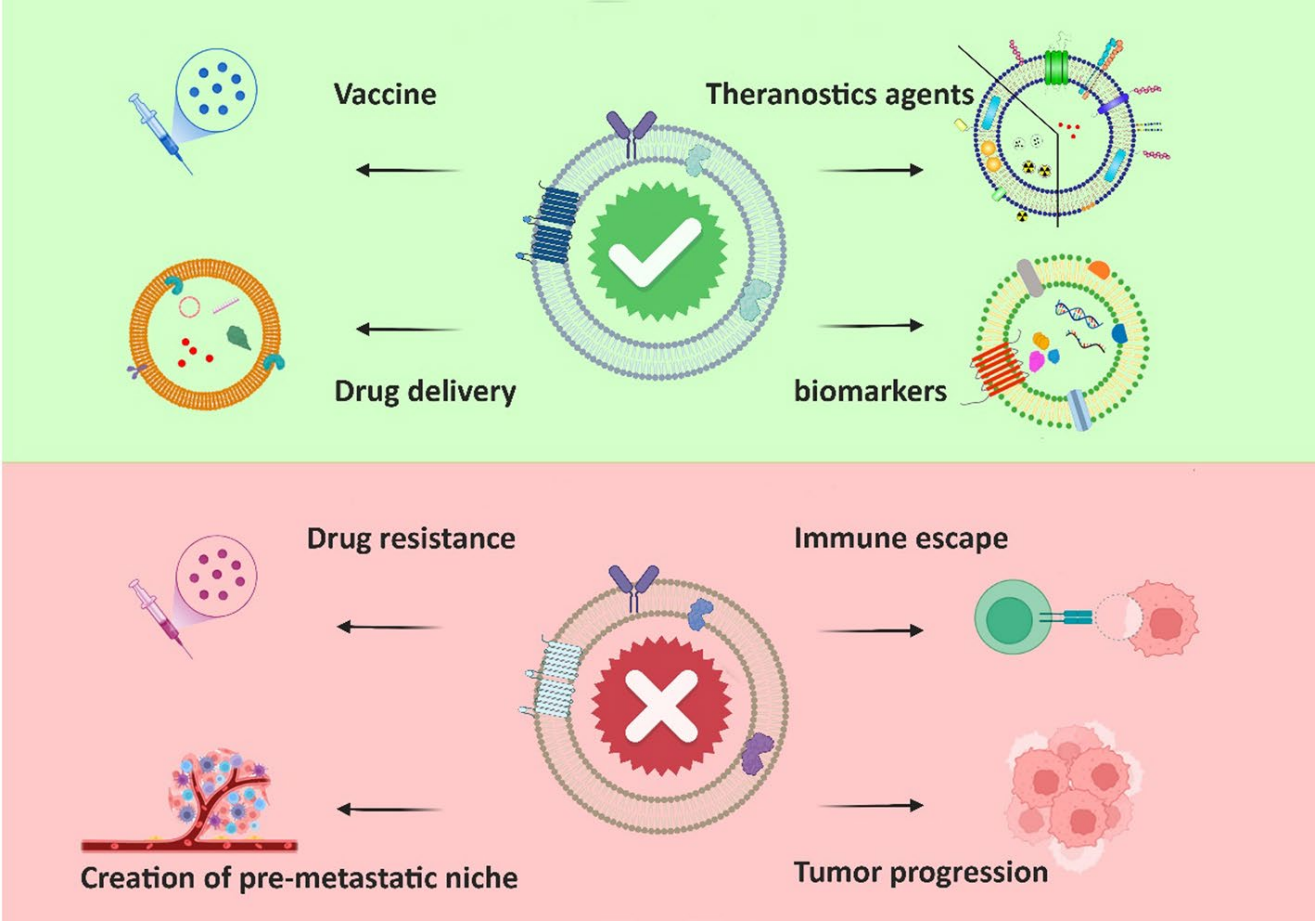

## Introduction

**Fig. 1:**
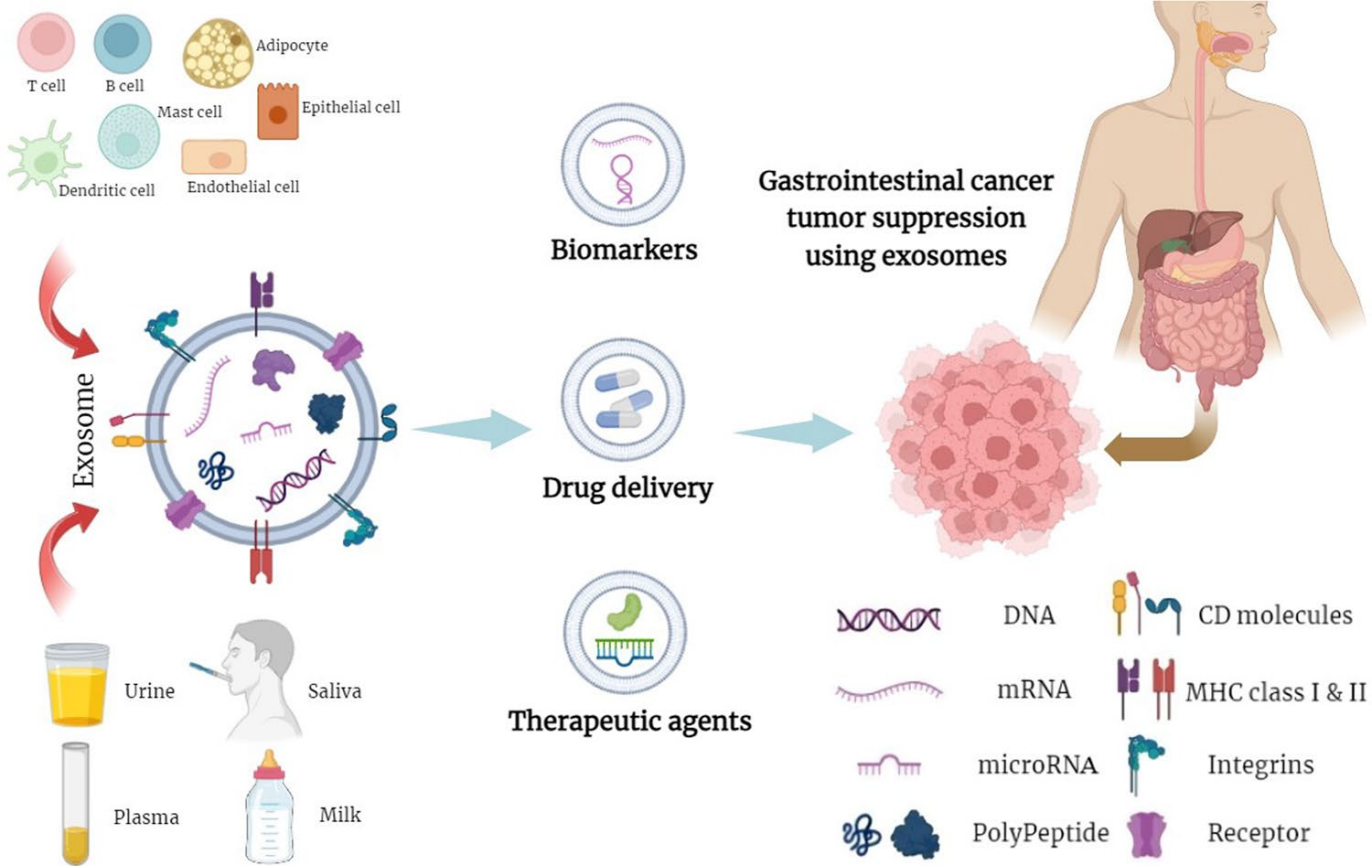
Schematic figure of the effect of exosomes on gastrointestinal cancer. Small extracellular vesicles are surrounded by a lipid bilayer membrane and are secreted by various types of cells, including cytotoxic T cells, B lymphocytes, platelets, dendritic cells (DCs), mast cells, adipocytes, neurons, endothelial and epithelial cells originate. Exosomes are isolated from sources such as saliva, plasma, milk, amniotic fluid, synovial fluid, urine, tears, nasal secretions and pleural effusion. Depending on the cell source and environmental conditions, the exosome content is different and may include DNA, RNA, polypeptide, CD molecules, etc., which have the potential to regulate the expression of various genes and interfere in cellular mechanisms. These characteristics can be used to carry drugs or therapeutic agents and also as biomarkers in gastrointestinal cancer

Based on size, extracellular vesicles are classified into three groups: exosomes (30–150 nm), microvesicles (100–1000 nm), and apoptotic bodies (> 1000 nm) [1]. Exosomes are extracellular vesicles that were first described by Harding et al. in 1983, and confirmed by Johnstone et al. in 1987 [2]. Extracellular vesicles are surrounded by a lipid bilayer membrane and originate from multivesicular bodies secreted by various types of cells. Exosomes are formed via endocytosis and can be derived from most mammalian cells, including cytotoxic T cells, B lymphocytes, platelets, dendritic cells (DCs), mast cells, adipocytes, neurons, endothelial cells, and epithelial cells (Fig. 1) [3]. Depending on the cellular source and the environmental conditions to which exosomes are subjected, the membrane content and composition are highly heterogeneous and dynamic [4]. The release of exosomes occurs both under physiological and pathological conditions. In addition to cells, exosomes are also isolated from sources such as bovine milk [5, 6] and are present in almost all body fluids, including saliva, semen, plasma, human breast milk, amniotic fluid, bronchoalveolar lavage, cerebrospinal fluid bile, synovial fluid, urine, tears, nasal secretions, and pleural effusions [3]. Depending on cellular origin, exosomes can contain different components such as proteins, nucleic acids, and lipids; because of this diverse composition, they have the potential to regulate the expression of various genes [7]. Initially, exosome production was mainly considered as part of a process to dispose of cellular waste products; however, various other functions emerged over the years [8]. For example, exosomes play a critical role in intercellular communication (in addition to established mechanisms such as direct cell–cell contact and transfer of secreted molecule) [9], and can be used in gene therapy and drug delivery [10]. For example, exosomes derived from raw bovine milk were tested as carriers of extracellular RNAs aimed at delivering hsa-miR148a-3p to liver (HepG2) and intestinal (Caco-2) cell lines. The results showed that this cost-effective source can be used as a nanocarrier of functional microRNAs (miRNAs) in RNA-based therapy [6]. Compared to viral vectors/liposomes, exosomes are less immunogenic and have the ability to cross major physiological barriers such as the blood–brain barrier, making them an attractive option as biomarkers and therapeutic agents [11]. Furthermore, exosomes are vital in antigen presentation and immune system activation [7], properties that can be utilized in vaccination [8]. Effective application of exosomes has also been reported in the diagnosis and therapy of several diseases, particularly cancers [12]. Exosomes can affect cancer progression via various mechanisms, including angiogenesis, modulation of immune response, metastasis, drug resistance, and tumor growth or development [1, 3]. Gastrointestinal (GI) cancers are among the deadliest cancers and can develop in the upper parts of the GI tract, e.g., the esophagus and stomach, or in other organs such as the liver, pancreas, small intestine, and colon [13]. Gastric malignancy is the fourth most prevalent cancer and the most common cause of cancer-related deaths in recent years.. Despite significant advancements in various treatment strategies such as chemotherapy, radiotherapy, immunotherapy, and surgery, tumor metastasis and/or recurrence are still the most common causes of cancer death, which is due to poor prognosis in this field [14]. Based on the current literature, timelier (i.e., early) diagnosis and increased knowledge of risk factors would significantly benefit cancer survival. Thus, there is an urgent need for new non-invasive diagnostic methods to improve early cancer diagnosis and prognosis [15]. For example, the survival rate of patients with colorectal cancer (CRC), one of the most common cancers with high mortality rate, would be significantly increased by early diagnosis [16]. As indicated, exosomes have been reported as a novel approach in the diagnosis and treatment of cancers [17], especially in gastrointestinal cancers [16], and their role as diagnostic biomarkers or drug carriers is well studied [12]. Exosomes containing lncRNA RPPH1, derived from tumor cells, were shown to be important in early diagnosis of CRC [18]. In another study, the disbalance of exosomes containing miR-217 was considered a diagnostic biomarker in gastric cancer [19]. Recent work indicated that exosomes containing miR-9-3p or miR-21 can act as biomarkers for early detection of metastasis in liver cancer (HCC), a malignant cancer that has no specific symptoms in the early stages [20]. In this review, we describe the functional and mechanistic roles of exosomes in the development and progression of gastrointestinal cancers. In addition, we discuss the potential clinical and biomedical applications of exosomes in gastrointestinal cancers.

## Exosome biogenesis, functions, mechanisms, and applications

### The biogenesis of exosomes

**Fig. 2:**
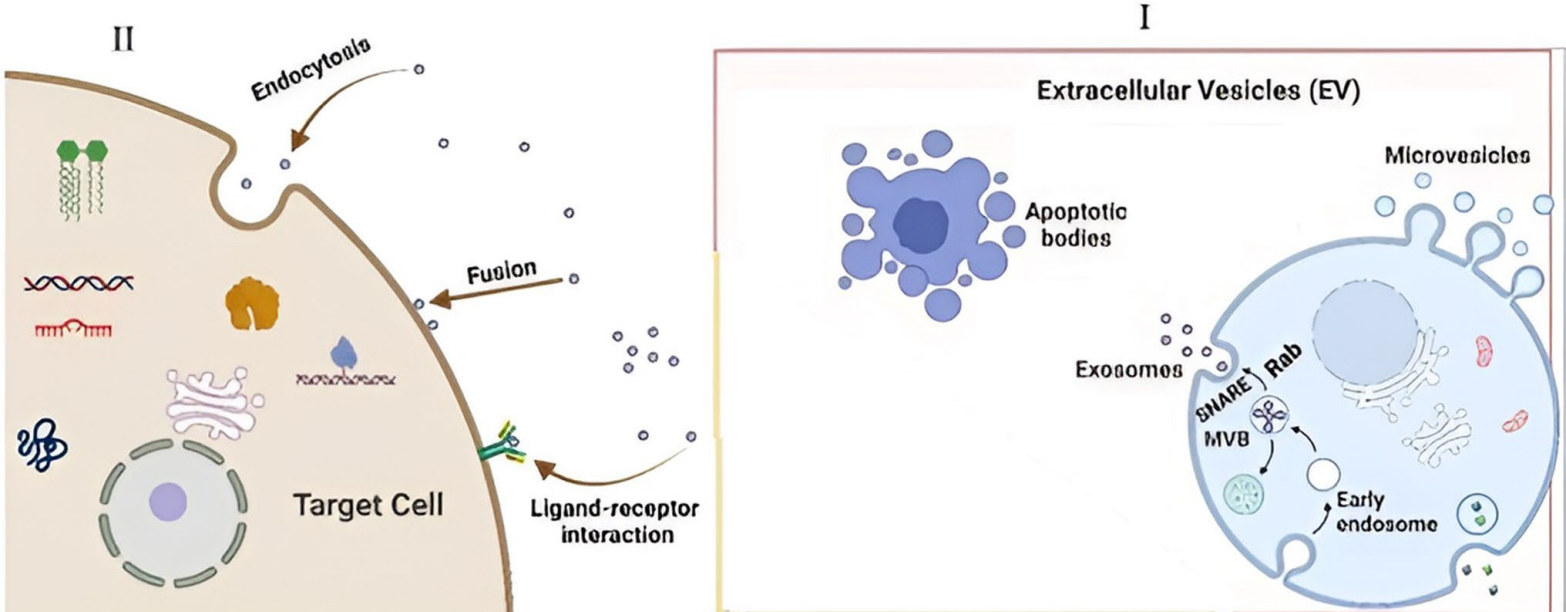
(I) The biogenesis and composition of exosomes. Biogenesis is divided into three stages: (i) endocytosis, (ii) endosome formation, and (iii) exocytosis. The endosome is formed by endocytosis and then transforms into a late endosome that contains multivesicular bodies (MVBs). MVBs secrete small vesicles (exosomes) to the extracellular membrane. (II) The released exosomes can then target cells through three major pathways: endocytosis, fusion, and ligand-receptor interactions

Exosome biogenesis starts with double invasion of the plasma membrane by endocytosis, followed by formation of the primary endosome, which matures into a secondary endosome or multivesicular body (MVB) (Fig. 2), which itself contains intraluminal vesicles (ILVs) [21]. The formed MVB has two possible fates: it can combine with lysosomes and destroy its cargo, or merge with the cell membrane and release its ILVs into the extracellular environment [22]. The stages of exosome formation in MVBs include the entry of specific lipids and proteins into the endosomal membrane as well as the entry of molecules into primary ILVs and the subsequent separation of ILVs. The exact process of cargo sorting is still unknown; however, two main mechanisms have been identified for the entry of the cargo into the exosome. The first depends on ‘endosomal sorting complexes required for transport’ (ESCRT), which takes place on the cytosolic side of the MVB membrane and recognizes transport, trans-Golgi network, and cell surface proteins; these proteins are then ubiquitinated and directed into the exosome. The second mechanism is independent of ESCRT, and relies on the lipid content of the endosomal membrane [23, 24]. Studies have shown that ESCRT components play an important role in the formation of MVB and ILV. This complex contains thirty different types of proteins, including those classified into ESCRT-0, -I, II, and -III [8], VPS4 (vacuolar protein sorting-associated protein) [25], VTA1 (vesicle trafficking 1) [21], and Alix (apoptosis-linked gene 2-interacting protein X) [26]. The main function of ESCRT is to include and sort specific components in ILVs that are supposed to be converted into exosomes [8]. The reaction of ESCRT-0 with phosphatidyl inositol triphosphate, located on the endosomal membrane, activates ESCRT-0 and binding to ubiquitination proteins, leading to the recruitment of ESCRT-II components. The involvement of ESCRT-1 and -II signals the beginning of budding towards the inside of the MVB. Near the bend of the membrane of the forming ILVs, ESCRT-II activates the components of ESCRT-3, which with its ATPase enzymes causes the separation of ESCRT subsets and vesicles. Recent studies reported that inhibition of the expression of specific ESCRT components, such as tumor susceptibility gene 101 (TSG101) or hepatocyte growth factor-regulated tyrosine kinase substrate (HRS), decreases the amount of exosome production and secretion [8, 20, 21, 27, 28]. As indicated, the second pathway of MVB formation is ESCRT-independent and relies on the lipid composition of the endosomal membrane. Thus, the formation, loading, and release of exosomes is highly dependent on ceramides produced as a result of sphingomyelinase activity [23, 29]. Other proteins involved in the process of exosome biogenesis include syndecan heparan sulfate proteoglycans and their cytoplasmic adaptor syntenin, sytenin1, syndecan-1, tetraspanins (CD9, CD63, and CD82), Ras-related protein GTPase Rab (Rab27a, Rab27b), and SNARE (soluble N-ethylmaleimide–sensitive factor (NSF) attachment protein receptor] [1, 23, 30].

### Functions of exosomes

Exosomes were traditionally considered as cellular waste. Recently, however, exosomes have been demonstrated to play various biological and pathological roles. Depending on their cellular origin, they can have different compositions and, therefore, fulfil distinct functions [15, 30]. Exosomes are present in most body fluids and can transport various types of cargo affecting cellular activities [20]. Exosomes can be related to various types of diseases. The evidence shown an increase in exosome secretion in cancer patients. Cancer cell-derived exosomes can play an essential role in tumorigenesis and tumor growth. They can also integrate with cells at specific locations, creating a pre-metastatic niche facilitating metastasis and cancer progression. In addition, exosomes have been shown to be important in cancer drug resistance, angiogenesis, and immune escape. Furthermore, because they transport various molecules such as nucleic acids and proteins [8], exosomes have been linked to the development of cancers [23, 31]. Some exosomes contain non-coding RNAs (miRNA let-7) and can cause cancer progression; e.g., exosomes secreted from human gastric cancer cell line can activate the AKT signaling pathway and increase proliferation [32]. Moreover, exosomes derived from liver cancer cells cause tumor growth by establishing communication between cancer cells and activating the hedgehog signaling pathway [33]. Thus, based on the available literature, it appears that specific exosome cargo could be crucial in cancer formation, and particularly in tumorigenesis [23, 34]. Understanding the effects of exosomes on cancer development and progression would help us to develop therapeutic strategies specifically aimed at impacting exosome formation, release, and receptor cell uptake. Exosomes are considered as a suitable option for gene therapy due to their ability to transfer nucleic acids, without activating the host's immune system or causing (cellular) toxicity [35, 36]. Recently, the diagnostic aspects of exosomes have also been investigated. Thus, different patterns of exosomal microRNAs and RNAs in patients and healthy people would allow the use of exosomes as diagnostic biomarkers; for example, reduced expression of exosomal miR_92 can be indicative of hepatocellular carcinoma and leukemia [37]. Exosomes also play a role in the transfer of various substances or cell signals involved in cancer progression (e.g., KRAS mutation in pancreatic cancer). Furthermore, exosomes can serve as drug delivery systems, considering their high half-life, ability to target specific cells, biocompatibility, and non-toxicity [8, 38]. With regards to the role of dendritic cells (DCs) in antigen presentation, exosomes can act as antigens for DCs, which in turn can activate the immune system against cancers and regulate immune responses. The ability of exosomes to promote or suppress cancers, and their effective role in immunotherapy should qualify exosomes as targets/tools of interest in novel therapeutic strategies in the near future. Some studies have already shown that exosomes derived from NSCLC tumors can help DC maturation by increasing the expression of Rab27a and upregulation of MHC, thereby promoting the proliferation of CD4 + T cells [20].

### Role of exosome in GI cancer progression

As indicated, exosomes can contribute to the development of cancer through various mechanisms, which we will discuss in the following sections.

#### Exosome and tumorigenesis

**Fig. 3:**
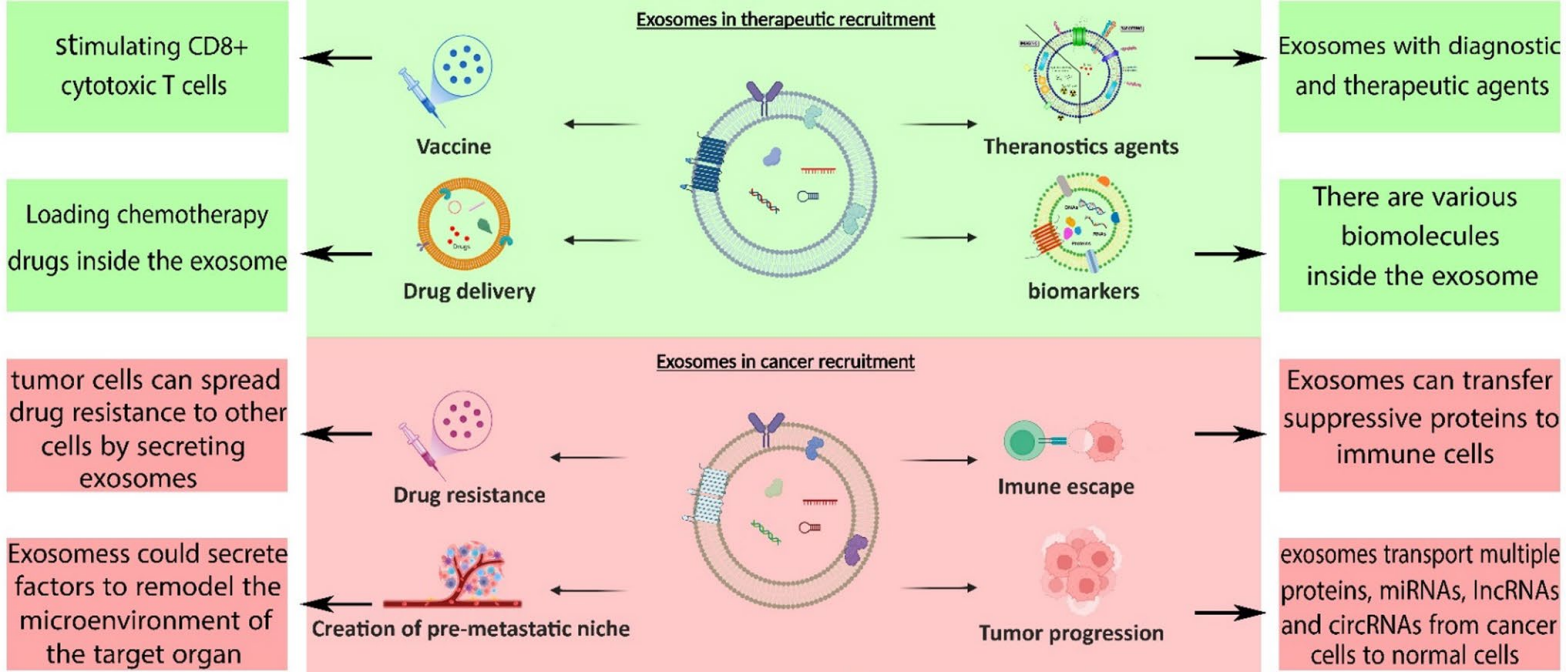
The roles of exosomes in cancer. Exosomes can be used as agents against cancer. For example, they can carry and deliver theranostic agents (diagnostic and therapeutic agents simultaneously in one platform), cancer diagnostic agents (important biomarkers), and conventional chemotherapy drugs. Conversely, exosomes may also cause tumorigenesis or cancer progression as destructive agents, and certain exosomes have been found to play a role in drug resistance, immune escape, tumor progression, and development of pre-metastatic niches

LncRNA HEIH, which is released by gastric cancer (GC) cells, can play a role in tumorigenesis. Exosomes secreted from a SGC-7901 cell line caused the propagation of BGC-823 and SGC-7901 cells via activation of the Akt signaling pathway [39]. Cancer exosome mirs have also been implicated in tumorigenesis. Importantly, exosomal miR biogenesis has been described, for example, this is the case for miR_Let7, which increases metastasis in GC [7, 14, 30, 40]. The role of exosomes in relation to cancer is summarized in Fig. 3.

#### Exosome and tumor growth

Both cancer- and microenvironment-derived exosomes can be involved in tumor growth. Cancer cells try to grow and survive through various mechanisms; the enhancing effects of exosomes on cancer cell growth have been widely reported in various cancers. For example, cancer cells can absorb exosomes that contain heat shock proteins (HSP), such as HSP90 and HSP70, which promotes their proliferation and inhibits apoptosis [41]. Colon cancer cell-derived exosomes containing the ΔNp73 gene are able to induce proliferation of recipient cells. Furthermore, tumor-derived exosomes are multiplied by mRNAs and eventually cause tumor growth in CRC. Other work showed that cancer-derived exosomes impact tumor growth of hepatocellular carcinoma (HCC) through the regulation of TAK1 expression. In addition, the loss of miR320a-containing exosomes derived from cancer-related fibroblasts (CAF) induced proliferation of HCC cells [23, 30, 42].

#### Exosomes and angiogenesis

Tumor growth and metastasis importantly rely on angiogenesis for supply of nutrients and oxygen. Exosomes act as mediators between tumor cells and vascular endothelial cells, and play a role in cancer progression as carriers of angiogenic factors. In addition, tumor-derived exosomes greatly affect metastasis through vascular remodeling [43]. They can also interfere with the integrity of endothelial cells; e.g., exosomal miR-105 increased vascular permeability and metastasis by reducing ZO-1 protein expression (44). It has been demonstrated that some exosome miRs are involved in the angiogenic process. For example, in CRC, miR-9 can promote angiogenesis and enhance endothelial cell migration by inhibiting the expression of suppressor of cytokine signaling 5 (SOCS5) [45]. Another study found that exosomes from colon cancers and pancreatic adenocarcinoma increase metastasis and angiogenesis by carrying tetraspanin 8 [46]. Thus, exosomes can affect angiogenesis in two ways, either by directly delivering angiogenic factors to endothelial cells or via exosomal miRNAs [7, 8, 20, 23, 30].

#### Exosomes and metastasis

Tumor metastasis involves cancer cells migrating to distant points and is one of the leading causes of cancer-related death. The higher the percentage of tumor malignancy, the more likely it is for cancer cells to invade and migrate to other organs and metastasize [14, 30]. Exosomes have been suggested to impact tumor metastasis in three main ways:

1. Tumor-derived exosomes can increase cancer cell invasion and metastasis by acting on the extracellular matrix (ECM).
2. Exosomes can loosen the tight connections between (endothelial) cells, which increases the penetration of tumor cells.
3. Exosomes can increase the metastasis and invasion of cancer by enhancing epithelial-mesenchymal transition (EMT) [47–50].

Recent studies have shown that GC cells can induce the penetration of peritoneal mesostromal cells (PMCs) by releasing exosomes containing Wnt3a. PMCs then invade the stomach wall and provide the basis for metastasis [51]. In addition, it was demonstrated that GC cell-derived exosomes carrying EGFR are transferred to liver cells and increase hepatocyte growth factor (HGF) by inhibiting miR-26a, thereby favoring metastasis [30]. Other work showed that exosomes harboring miR-221/222 stimulate GC cells to migrate [52]. Further supporting a role for exosomes in metastasis, gastrointestinal stromal tumor cells release exosomes containing protein tyrosine kinases to convert smooth muscle progenitor cells into a premetastatic site [42].

#### Exosomes and drug resistance

One of the most critical obstacles in cancer treatment is drug resistance; recent studies have indicated that exosomes may play a role in this process through various mechanisms. Tumor cells can spread drug resistance by secreting exosomes that may transfer proteins, miRNAs, and/or long non-coding RNAs to other (recipient) cells [20, 30]. Tumor-derived exosomes can also transfer multidrug resistance (MDR) by impacting the expression of multidrug resistance proteins (MRP) as determinants of cancer drug resistance [20, 42]. Recent studies reported that miRNAs of exosomes derived from cancer stem cells (CSC), including the highly expressed miR-210 in pancreatic CSC exosomes, can participate in the transfer of resistance to sensitive cancer cells [20].

#### Exosomes and immune escape

Exosomes can affect the formation, maturation, and anticancer activity of immune cells by transferring suppressive proteins. In addition, they can transfer DNA, mRNA, and/or miRNA, and increase cancer progression via reprogramming the function of the response cells [30]. For example, tumor cells induce apoptosis in T lymphocytes by releasing exosomes containing cell death receptors [14]. In addition, exosomal miR-24-3p can inhibit the proliferation and differentiation of T cells by silencing FG11 expression (30). Exosomes can also suppress cell differentiation (e.g., from myeloid to DC), reduce immune system activation, and facilitate immune evasion. A recent study showed that exosomes derived from NPC cells interfere with T cell function by regulating miRNAs [42]. Moreover, GC-derived exosomes play a role in regulating the immune system to promote development of GC. In fact, this regulation is done through Noncoding RNAs (ncRNAs). ncRNAs represent a substantial portion of the content within exosomes, and certain ncRNAs with biological functions are specifically packaged into these extracellular vesicles. Recent studies have unveiled the critical roles played by exosome-derived ncRNAs in the tumorigenesis, progression, and drug resistance of gastric cancer (GC). Moreover, the regulation of exosomal ncRNA expression levels has the capacity to either promote or suppress the advancement of GC [14].

### The application of exosomes

#### Exosomes as biomarkers

Exosomes have emerged as important diagnostic and prognostic biomarkers in several cancers (Table 1), and can be considered for many therapeutic purposes. In addition, tumor-derived exosomes can be utilized as vaccines in clinical and preclinical studies. Because exosomes are present in virtually all body fluids and may contain various bioactive molecules, it is much easier to detect cancer. Indeed, evaluating the expression of exosomal miRNAs has been used as a tool for diagnosing cancer progression in several cancers, including GC, in which lncRNA was identified as a new exosomal biomarker [42]. Several other exosomal miRNAs, i.e., miR 150-3p, miR-145-3p, miR-139-3p, and Let-7b-3p, have emerged as diagnostic biomarkers in colon cancer [53]. Urinary exosomal lncRNA, which includes PCAT-1, MALAT1 and SPRY4-IT1, serves as a biomarker for the detection and recurrence prediction of bladder cancer [12]. Exosomal miRNAs can also indicate metastasis in GC (e.g., miR-101-3p, miR-10b-5p, and miR-143-5p) [14]. In addition to miRNAs and lncRNAs, exosomes may contain other cargo helpful for cancer diagnosis; for example, the exosomal protein glypican-1 (GPC-1) is a biomarker for the diagnosis of pancreatic cancer [3].

**Table 1:** Exosomes as biomarkers

Exosome biomolecules	biomolecules	Type of cancer	References
Exosome proteins	FZD-10	gastrointestinal	[59]
	CD41	gastrointestinal	[60]
	(GPC-1)	pancreatic, breast, and colorectal cancer (CRC)	[58]
	CD9	CRC	[61]
	CD147	CRC	[61]
	MIF	pancreatic ductal adenocarcinoma (PDAC)	[62]
Exosomal lipids	linoleic acid (LA), γ-linolenic acid (GLA), and arachidonic acid (AA)	colon adenocarcinoma Caco-2	[58]
	Eicosapentaenoic acid (EPA) and Alpha-linolenic acid (ALA)	human colon epithelial cells (HCEC-1CT)	[58]
Exosome RNAs	miR-10b	CRC	[75, 76]
	miR-21-3p and miR-769-3p	Metastatic CRC	[77]
	miR-1246	CRC	[78]
	miR-21	hepatocellular carcinoma	[79]
	hTERT mRNA	pancreatic cancer	[80]
	miR-21	gastric cancer (GC)	[81]
	ZFAS1	GC	[82]

#### Exosomes and drug delivery

Exosomes can potentially be used as drug carriers to treat various diseases. Due to their characteristics such as biocompatibility and biodistribution, they are suitable carriers for various substances. For example, the transfer of exosome-derived siRNA can cause specific gene silencing and induce cancer cell death [42]. Exosomes have several advantages over some other carriers (e.g., Liposomes and synthetic nanocarriers), including a higher drug delivery efficiency and the ability to prevent macrophage phagocytosis with limited immunogenicity. Thus, exosomes increase the half-life of the drug, do not cause toxicity, are accurate in cell targeting, and promote endocytosis, all of which facilitates the drug delivery process. Exosomes carrying nucleic acids, such as the highly expressed let-7 and several other miRs, can act as tumor suppressors. For example, exosomal miR-335-5p can reduce the size of liver tumors [54], and miR-145-5p (by activating the Smad3 pathway) inhibits pancreatic cancer cells multiplication [55]. In addition, chemical anticancer drugs, such as paclitaxel (PTX) and doxorubicin, can be carried by exosomes as well [12, 56]. Considering exosomes can identify specific cells, exosomal delivery of therapeutic compounds can be more effective and precise than with other biocarriers such as liposomes. Therefore, exosome therapy is not only suitable for diagnostic analysis but also promising in cancer treatment [12].

## Exosomes as a biomarker for the diagnosis of gastrointestinal malignancies

### Exosome proteins as biomarkers in GC

Some of the housekeeping protein markers used to distinguish exosomes from other extracellular vesicles include tumor susceptibility gene 101 (TSG101), ALG-2-interacting protein X (ALIX), CD63, CD81, and HSP70; these proteins are important in the biogenesis of exosomes [57]. In this section, however, we will discuss exosomal protein biomarkers that have been specifically linked to gastrointestinal cancers. Frizzled family proteins are important Wnt pathway receptors and can contribute to the development of cancer stem cells. In particular, Frizzled-10 (FZD-10) plays a role in gastrointestinal cancers and has been detected on the surface of exosomes derived from cells of these cancers [58]. Thus, a recent study utilizing Au nanoparticles (surface-functionalized with a FZD10 protein primary antibody) and a transmission electron microscopy (TEM) grid detected FZD10 protein on the surface of gastrointestinal cancer cell-derived exosomes [59]. Others studied exosomes isolated from the blood of cancer patients and healthy donors using atomic force microscopy (AFM) to detect exosomes positively expressing the surface marker CD41 [60]. The GPC-1 protein has been identified as an exosomal marker in pancreatic, breast, and colorectal cancer [58]. In addition, CD9 and CD147 have been found to be highly expressed in exosomes isolated from the serum of CRC patients. It should be noted, however, that exosomal surface expression of CD47 decreased after tumor surgery in these patients [61]. Costa Silva et al. demonstrated that pancreatic ductal adenocarcinoma (PDAC)-derived exosomes play a role in the development of hepatic pre-metastatic niches. Further investigations showed that these exosomes highly expressed macrophage migration inhibitory factor (MIF). Based on these results, the authors suggested that MIF positive exosomes could serve as biomarkers to indicate the development of PDAC liver metastasis [62].

### Exosomal lipid profiles as biomarkers

Lipid metabolism is important in the carcinogenesis of many cancers, especially CRC [63–65]. For example, lysophosphatidylserine abundance is significantly increased in colon cancer tissues as compared to surrounding normal tissues [66]. Pro-inflammatory stimuli promote a microenvironment favoring cancer development [67]. Lipidomic evaluation of tissue inflammation revealed a significant relationship between the change in the structure of membrane lipids and the development of inflammation, implying some of these lipids associated with inflammation can serve as biomarkers [68, 69]. One study found that the lipid structure of the exosomal membrane is affected by the cells from which exosomes are extracted. In an in vitro study related to prostate cancer, it was demonstrated that the lipid composition of exosomes is similar to the membrane structure of cancer cells from which they were derived [58]. Recently, the lipid profile of exosomes extracted from human colon adenocarcinoma Caco-2 and human colon epithelial cells (HCEC-1CT) was evaluated. Exosomes derived from Caco-2 cells exhibited a distinct lipid profile, with high amounts of linoleic acid (LA), γ-linolenic acid (GLA), and arachidonic acid (AA). In addition, exosomes from HCEC-1CT cells showed higher amounts of omega-3 fatty acids, including eicosapentaenoic acid (EPA) and alpha-linolenic acid (ALA), as compared to those derived from Caco2 cells. The results indicated a proinflammatory role of omega-6 in CRC tumorigenesis, as evidenced by high n-6/n-3 and AA/EPA ratios in Caco2 cells [69, 70].

### Exosomal RNAs as biomarkers

Several studies have investigated the effects of RNAs delivered by exosomes in tumorigenesis and cancer spread. For example, long non-coding (Lnc) RNAs such as Lnc-sox2ot, Lnc-h19, and LncRNA-ARSR, which have been identified in exosomes, are involved in the progression of tumors [71–74]. Dysregulation of miRNAs delivered by exosomes has been associated with gastrointestinal malignancies. MiR-10b-containing exosomes, which are secreted in the CRC tumor microenvironment by cells like cancer-associated fibroblasts (CAF), can increase the expression of transforming growth factor-beta (TGF-β) and smooth muscle (SM) α-actin, ultimately promoting the growth of CRC cells [75, 76]. Exosomal delivery of miR-21-3p and miR-769-3p has been shown to play a role in CRC metastasis to the lung through the activation of fibroblasts in the tumor microenvironment and lung tissue; this process occurs through the formation of premetastatic niches, and the secretion of these types of exosomes increases following p53 R273H mutation [77]. Cooks et al. reported that miR-1246-enriched exosomes are secreted from CRC cells that have mutations in p53, and contribute to CRC progression and metastasis. Therefore, these exosomes are important in the diagnosis of this type of CRC [78]. MiRNA-21-containing exosomes may be involved in the development of hepatocellular carcinoma through the conversion of normal hepatic stellate cells (HSCs) to CAFs. This cellular transformation (associated with tumorigenesis) is induced by exosomal targeting of phosphatase and tensin homologue (PTEN), and the subsequent secretion of factors such as TGF-β, fibroblast growth factor-2 (FGF-2), and endothelial growth factor (VEGF) [79]. Transcription of enzyme telomerase (hTERT mRNA) delivered by exosomes can cause the transformation of normal fibroblasts into telomerase-positive cells; this affects the microenvironment of pancreatic cancer, which can be important in terms of cancer metastasis [80]. MiR-21 has been identified in exosomes associated with GC cells. Considering it regulates the important PTEN/PI3K/AKT signaling pathways, this micronucleic acid can be effective in apoptosis inhibition and cisplatin resistance [81]. LncRNA ZFAS1 is another important RNA identified in GC-derived exosomes, and has been shown to impact MAPK signaling, EMT, cell cycle progression, as well as cancer growth and metastasis. Based on the results of this study, it is concluded that ZFAS1, plays a role in GC progression and metastasis. Therefore, it has been suggested that ZFAS1 can be considered as a diagnostic and prognostic biomarker in this cancer [82]. In addition to preclinical studies, several clinical trials evaluating exosomes as biomarkers and diagnostic factors for gastrointestinal cancers have been conducted (summarized in Table 2).

**Table 2:** Clinical trial studies evaluating exosomes for the diagnosis of gastrointestinal cancers

ClinicalTrials.gov ID	Study type	Observation	State	Phase	Description	References
NCT03874559	Observational	Diagnostic test: blood draw**.** Serum samples will be obtained from each patient enrolled	Active	–	characterize exosomal biomarker levels in patients with locally advanced rectal cancer undergoing neoadjuvant chemoradiation therapy	[152]
NCT05427227	Observational	Device: EV-array detection collect peripheral blood sample of 500 GI patients at treatment baseline	Active	–	All samples will be processed by exosomes proteome detection to explore the efficacy and mechanism of anti-HER2, immunotherapy and anti-CLDN18.2 of gastrointestinal cancer	[153]
NCT02393703	Observational	Blood and tissue from patients with pancreatic cancer will be compared with blood and tissue from patients with noncancerous pancreatic disease	Recruiting	–	Investigation assessing whether exosome activity has a connection to disease recurrence and outcomes in patients	[154]
NCT04394572	Observational	Identification of new Diagnostic protein markers for Colorectal Cancer in Circulating tumor exosomes	Active	–	Exosomes will be isolated from sera of patients and their number, size and protein composition will be characterized	[155]
NCT04227886	Observational	Identification of biomarkers (from patient tumor biopsy and peripheral blood samples before neoadjuvant therapy) for the prediction of the response and toxicities to neoadjuvant therapy to stratify patients and optimize treatment strategy	Active	–	Study on predictive biomarkers of neoadjuvant chemoradiotherapy for rectal cancer	[156]
NCT03821909	Observational	Acquisition of portal venous circulating tumor cells and exosomes from patients with pancreatic cancer by endoscopic ultrasound: a prospective study	Active	–	Exploration of the feasibility and safety of sampling portal venous blood via endoscopic ultrasound (EUS), and detect portal venous circulating tumor cells (CTCs) and analyze mRNA markers of exosomes by RNA-seq	[157]
NCT01779583	Observational	Evaluation of circulating exosomes in advanced gastric cancer patients: A prospective observational Study	Completed	–	Evaluating the prognostic and predictive value of gastric cancer exosome levels in plasma and characterize the molecular profile of gastric cancer-derived exosomes	[158]
NCT03581435	Observational	A study of circulating exosome proteomics in gallbladder carcinoma patients	Active	–	collection of blood and tumor tissue samples for further proteomics studies. exosomes from blood specimens will be isolated and purified by sucrose gradient ultracentrifugation	[159]
NCT04852653	Observational	Test the detection of tumor extracellular vesicles (EVs) in liquid biopsy as a reliable marker	Recruiting	–	Detecting tumor extracellular vesicles (EVs) in liquid biopsies to identify the response of rectum cancer to neoadjuvant treatment	[160]
NCT04523389	Observational	Contents of circulating extracellular vesicles: Biomarkers in colorectal cancer patients	Active	–	circulating exosomes derived from tumors contain markers, including specific miRNAs, that could be used as biomarkers of early prognosis	[161]
NCT05625529	Observational	The exoLuminate study for early detection of pancreatic cancer using blood samples	Recruiting	–	Eligible subjects will be evaluated using the ExoVerita™ assay through blood donation(s) at specified time intervals. Overall, this study poses minimal risk to study subjects	[162]
NCT03711890	Interventional	Imaging and detection of micrometer Sized early Stage pancreatic cancer by Using endoscopic ultra-high resolution optical coherence tomography (OCT) using resected pancreatic specimen	Recruiting	Not applicable	Evaluation of the accuracy of the optical coherence tomography (OCT)-based diagnosis compared to the pathological diagnosis or the cancer cell-derived exosomes test from the blood sample	[163]
NCT05575622	Observational	Clinical study for combined analysis of CTC and exosomes on predicting the efficacy of Immunotherapy in patients with hepatocellular carcinoma	Recruiting	–	CTC PD-L1 imaging, exosomal PD-L1 protein detection, and exosomal LAG-3 protein detection to identify the functional marker profiles of immunotherapy in peripheral blood of HCC patients	[164]
NCT06278064	Case–control	Quantitative investigation of the amount of protein in exosomes from plasma samples of people with esophageal and stomach cancer and the control group	Recruiting	–	Find specific protein biomarkers indicating tumors of the upper gastrointestinal tract in the early stages	[165]

The information was obtained on February 5, 2024 at 17:26

## Utilizing exosomes as therapeutic agents in cancer treatment

**Fig. 4:**
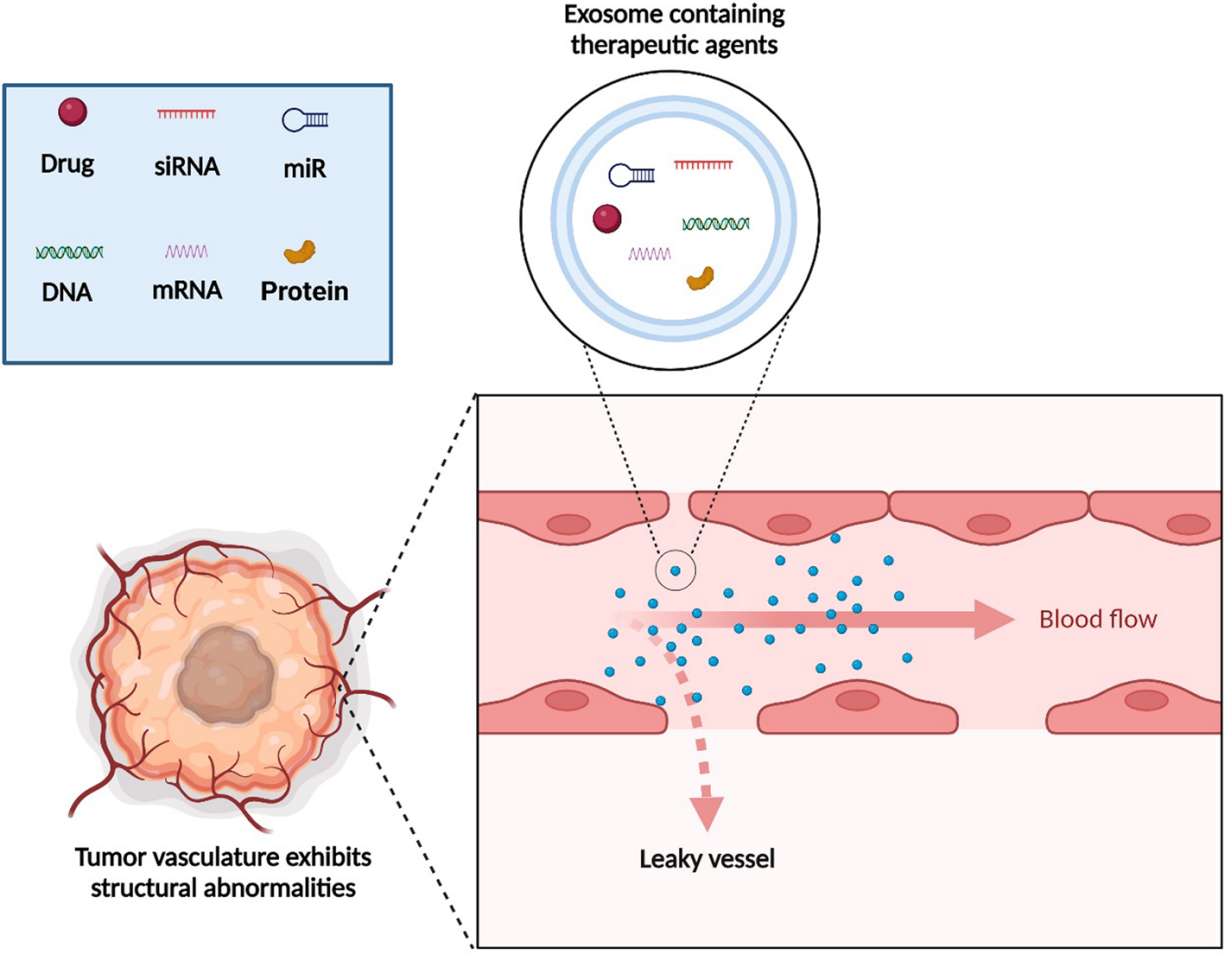
Exosomes containing therapeutic agents. Depending on the cellular source, exosomes can contain various therapeutic agents. Exosomes can effectively reach tumor cells through surrounding abnormal blood vessels, and have been utilized as a therapeutic approach against cancer

The role of exosomes as therapeutic agents depends on their original parental cells [83]. The most commonly used exosomes in cancer treatment are those secreted by mesenchymal stem cells (MSCs), DCs (dexosomes), and cancer cells [84]. Several studies have focused on exosomes derived from these sources, revealing that exosomes are frequently involved in cancer progression [84]. Exosomes have been employed as carriers of therapeutic agents, especially for the targeted delivery of small molecules. Their small size allows exosomes to penetrate the tumor tissue through the enhanced permeability and retention (EPR) effect [85] (schematic representation in Fig. 4). In several recent studies, exosomes have been designed and evaluated as theranostic nanostructures [86, 87]. Due to favorable characteristics such as excellent biocompatibility, high effectiveness, and minimal immunogenicity, exosomes are considered suitable nanocarriers for drug delivery cancer-related studies [88]. For example, exosome-delivered drugs such as doxorubicin (Dox) reduce cytotoxicity in sensitive body organs [89]. In addition, several studies confirm the efficiency of exosomes in drug delivery against gastrointestinal cancers. Pascucci et al. designed a treatment method based on MSC-derived exosomes carrying paclitaxel (PAC) in the tumor microenvironment. They demonstrated that exosomal release increased the anti-proliferative activity of PAC, effectively reducing the proliferation of cancer cells in pancreatic adenocarcinoma [90]. Others investigated the effectiveness of exosomes carrying anti-miR-214 in reversing chemoresistance to cisplatin in GC. This in vitro, showed that the exosomes could sensitize GC cells to cisplatin [91].

Exosome scaffold proteins have also emerged as promising molecules; for instance, they can be applied to improve the recognition of tumor cells by the immune system. For example, an exosome scaffold protein characterized from cloned cancer exosomes contained SIRP α (signal regulatory protein α), an antagonist of CD47 on tumor cells that has been proposed as a therapeutic tool to increase tumor cell phagocytosis by bone marrow-derived macrophages. In vivo studies show that tumor growth is reduced following this phagocytosis. In addition, protein delivery through exosome scaffold proteins is more effective as compared to that via protein-scaffold-based nanocages such as ferritin-SIRP α [92]. Another study investigated exosomes isolated from A33-positive LIM1215 cells and, after Dox loading, surface-functionalized with superparamagnetic iron oxide nanoparticles (SPIONs) coated with A33 antibody to target CRC. The findings revealed that the A33Ab-superparamagnetic nanoparticle-Exo/Dox platform can effectively prevent the growth of colon cancer cells [93]. Several ongoing clinical trial studies evaluating exosomes for gastrointestinal cancer treatment are listed in Table 3.

**Table 3:** Clinical trial studies based on exosomes for the treatment of gastrointestinal cancers

ClinicalTrials.gov ID	Study type	Intervention	State	Phase	Description	References
NCT01294072	Interventional	Plant exosomes containing curcumin	Active	I	Investigate the ability of plant exosomes to deliver curcumin more effectively to normal colon tissue and colon tumors	[166]
NCT03608631	Interventional	mesenchymal stem cell (MSC)-derived exosomes loaded with siRNA against KrasG12D (iExosomes) in metastatic PDAC patients with KrasG12D mutation	Recruiting	I	Evaluation of the optimal dose and side effects of mesenchymal stromal cell-derived exosomes containing KrasG12D siRNA (iExosomes) in treating participants with metastatic PDAC with KrasG12D mutation	[167]
NCT05375604	Interventional	Study of exoASO-STAT6 (CDK-004) in Patients with advanced hepatocellular carcinoma (HCC) and patients with liver metastases from either primary gastric or colorectal cancer (CRC)	Active	I	Study of macrophage reprogramming agent, exoASO-STAT6 (CDK-004) in Patients With advanced hepatocellular carcinoma (HCC) and Patients With liver metastases from either primary gastric or colorectal cancer (CRC)	(168)

The information was obtained on February 5, 2024 at 18:15

## Exosomes as biological drug carriers

In recent years, exosomes had a significant impact on the diagnosis and prognosis of numerous diseases, including diabetes, Parkinson’s, Alzheimer’s, cancer, and infectious diseases, as a result of the advancements made and the enormous increase in the number of various types of drug carrier systems available for use in clinical settings. The last several decades have seen increased interest in nanoparticles (e.g., polysomes, micelles, and liposomes) as drug carriers in clinical research. These nanoparticles are characterized by few undesirable side effects, a variety of medicinal substance delivery capabilities, large drug encapsulation, high efficacy, as well as low toxicity, and have the capacity to maintain drug concentrations, prevent drug degradation, interact with their biological environment, and increase drug absorption of the desired tissue. Exosomes are nanoparticles produced by cells that can outperform these ‘traditional’ nanocarriers. If these delivery systems are carefully developed in accordance with the target and route of administration, they could address some of the problems associated with the delivery of active molecules, such as peptides, proteins, genes, and oligonucleotides.

### Targeted delivery

High toxicity, multiple drug resistance, non-specific targeting, and poor stability are typical examples of the drawbacks encountered in drug delivery research [94]. Using immature DCs, Alvarez et al. were the first to show that exosomes can deliver medicines in a targeted manner [95, 96]. Exosomes can protect drugs from breakdown by the extracellular environment and are crucial in both physiological and pathological processes. Compared to other pharmaceutical systems (e.g., liposomes, lipid nanoparticles, viral vectors), exosomes have benefits to ensure efficient delivery, including better biocompatibility, lower immunogenicity and cytotoxicity in normal tissues, increased stability due to surface expression of CD55 and CD59, small size, the ability to cross the blood–brain barrier, high specificity for binding to the target cell, and a longer half-life. Additionally, Additionally, exosomes rely on a natural mechanism for the transport and delivery of specific drugs [97–99]. Although inconsistent results can be obtained by variations in the cells of origin, separation techniques, or specific protein/lipid surface profiles, exosomes generally perform better than conventional synthetic drug administration techniques [100, 101].

The cellular resistance to therapeutics is one of the major problems in treating gastrointestinal (GI) cancer. Despite the fact that the precise mechanisms underlying drug resistance are still incompletely understood, a number of contributing factors have been identified. Exosomes have been linked to targeting GI cancer invasion, angiogenesis, and treatment resistance [102–104]. Thus, exosomes can mediate endocytosis of specific medications into cancer cells [58], and can be genetically modified to express peptides or ligands on their surface to facilitate this process. Such modifications improve targeting and specificity by transferring certain exosome receptors and shortening the time it takes for exosomes to reach the therapeutic concentration in the intended tissues, collectively resulting in enhanced drug performance and better therapeutic effects [105]. Exosomes released by MSCs from healthy tissue may slow the growth of tumors by obstructing signaling pathways involved in oncogenic reprogramming. On the other hand, exosomes generated from tumor cells can cause malignancy and subsequent cancer of recipient cells [106]. Other work further supported the notion that targeted exosomes can be employed as an effective drug delivery method, demonstrating HER2 + cells take them up more readily than HER2- cells [107]. One of the main treatments for advanced stomach cancer is chemotherapy, Of course, it can also face challenges. The requirement for high dosages, low therapeutic indices, and maximum therapeutic concentrations, in addition to side effects, sensitive immunological responses, and the presence of mucosal physiological barriers represent some of the therapeutic challenges. It has been well documented that the necessary high medicine dosages can lead to drug resistance [99]. Exosomes fulfil a vital function in the detection and treatment of stomach cancer [108]. Exosomal circRNAs are highly promising therapeutic agents with anticancer effects, as indicated by their roles in controlling tumor growth, metastasis, angiogenesis, metabolism, and dissemination of GI cancers [109]. The development of targeted therapy has been considered for the treatment of gastric cancer; thus, gastric cancer exosomes have been reported to have high target efficacy, albeit with relatively low efficiency. Tian et al. developed a straightforward technique to produce high-performance gastric cancer hybrid exosomes as a possible drug carrier for targeted therapy of gastric cancer (HGCE). In vitro and in vivo studies demonstrated that Dox-loaded HGCE (Dox/HGCE) exhibited good anticancer efficacy as well as high and specific activity for gastric cancer cells (SGC 7901), indicating the therapeutic potential of this delivery system [110].

The importance of exosomes in GC suggests they could represent therapeutic targets. Proton pump inhibitors (PPIs) regulate the HIF-1-FOXO1 axis to stop stomach cancer from spreading. A high dose of PPI can inhibit GC malignancy and control the microenvironment surrounding the tumor. PPIs improve the effects of anticancer medications in GC cells by reducing stomach acid production. PPIs may be helpful as a therapeutic strategy for the treatment of GC considering they inhibit GC cells from releasing exosomes and prevent them from producing CAFs [111, 112]. Another study used exosomes as nanocarriers to transport circDIDO1 to GC cells; results showed that circDIDO1 can counterbalance the effects of miR-1307-3p overexpression in GC by serving as a miRNA sponge to stimulate SOCS2 expression, which in turn prevents the proliferation of GC cells [113]. Hosseini et al. looked at the targeted administration of Dox-loaded HEK293-derived exosomes functionalized with an anti-nucleolin (AS1411) aptamer for the treatment of CRC. The results showed that this functionalization markedly enhanced the binding affinity and uptake rate in nucleolin-positive cancer cells, suggesting Dox loading of AS1411-functionalized exosomes could serve as a potential cancer treatment approach in clinical settings [114]. Bagheri et al. looked at the potential of Dox-loaded exosomes produced by MSCs as a versatile tool for treating CRC and platform for therapeutic use. In vitro data demonstrated that DOX@exosome-apt delivers Dox to MUC1-positive cancer cells in a highly effective manner. Additionally, a single intravenous dose of DOX@exosome-apt significantly suppressed tumor growth as compared to free Dox in an in vivo study utilizing BALB/c mice and the C26 ectopic model (mouse colon cancer). Ex vivo fluorescence imaging further validated the beneficial biodistribution of DOX@exosome-apt by showing higher tumor accumulation and quicker liver clearance when compared to DOX@exosome and free Dox. Thus, MUC1 aptamer-functionalized exosomes can be used therapeutically to deliver Dox to colon cancer in a flexible and safe manner [102].

### Co-delivery therapy

According to recent studies, gene therapy or a cocktail of medications can be used to more effectively target pathways associated with cancer [115, 116]. Natural therapies can improve sensitivity to chemotherapy and strengthen immunity because they are risk-free and rarely cause harm. Widely biodistributed medications show both beneficial off-target effects and anticancer consequences. Combination drug therapy has been shown to be effective because the suppression of numerous mechanisms or junctions leads to the activation of a single mechanism. The benefits of combination therapy are supported by clinical trials showing synergistic effects [117–119]. Accumulating evidence suggests that combining (natural) chemotherapy sensitizers with chemotherapeutic agents can reduce drug-associated side effects and battle multidrug resistance (MDR) [120]. Co-delivery of various medications via a drug carrier may improve treatment for malignancies by synchronizing medication exposure and promoting synergistic pharmacological activity in tumor cells [121]. In addition, this approach allows for optimal loading capacity, stability, release kinetics, biocompatibility, and tumor targeting [122], and may effectively reduce toxicity, minimize adverse drug reactions, and overcome MDR, which is a significant obstacle to the long-term efficacy of chemotherapeutic agents. Furthermore, co-delivery systems can benefit controlled release in cancer treatment protocols, decreasing side effects of prescribed medications, and improving treatment effectiveness [123]. Selecting appropriate nanocarriers for efficient encapsulation of natural active substances and chemotherapy drugs is a significant issue [124]. Liposomes, micelles, nanoparticles, and inorganic nanoparticles are just a few of the co-delivery systems that have become available as a result of the advent of nanotechnology to battle tumor MDR. To prevent MDR, these nanocarriers are co-loaded with chemotherapeutics and natural products, limiting drug efflux and/or enhancing intracellular drug accumulation in either an active or passive manner. Understanding the features of the individual carriers, which all have distinct nanostructures, materials, and preparation procedures, will help with the design of co-delivery nanocarriers [125]. Ideally, these co-delivery systems should be able to encapsulate hydrophobic as well as hydrophilic medications, and transport both conventional chemotherapies and cellular regulatory molecules like nucleic acids [126]. Despite the significant advancements in nanotechnology, there are still a number of issues that need to be resolved in order to create the ideal drug delivery system. These include those related to encapsulating drugs with a variety of solubilities and physicochemical properties, increasing drug concentration in tumor tissues, and controlling their sequential drug release [127]. Theoretically, considering multiple cargo delivery capabilities of natural intercellular delivery systems would qualify exosomes as ideal nanoplatforms for the development of novel integration techniques [128]. Surprisingly, only a relatively small number of papers have reported on exosomes as co-delivery systems. Qi Zhan et al. described a novel combination gene/chemistry anticancer method in which blood exosomes were developed as a nanoplatform for the targeted and effective delivery of hydrophobic medicines and nucleic acids to tumor cells. This study demonstrated that these vesicles could efficiently and flexibly transport hydrophobic drugs like Dox and cholesterol-modified miR-21i by fully utilizing their original lipid bilayer structure. The addition of L17E peptides maximized the effectiveness of cargo delivery by hastening the endocytic absorption and endosomal egress of exosome-encapsulated payloads. This co-delivery nanosystem was able to preferentially accumulate in tumors because of the clusters of superparamagnetic nanoparticles and nanoscale size. In mice carrying the U87 gene that were systemically fed D-Exos/miR21i-L17E, tumor suppression was significantly enhanced with only moderate side effects. This is the first example of the use of blood exosomes in combined cancer chemo- and gene-therapy; by substituting a therapeutic medication combination and considering a range of tumor suppressor pathways, this constructed exosome-based nanosystem could be considered for a wide range of medicinal applications [129]. Recent work proposed a viable way to overcome drug resistance in CRC and boost the effectiveness of cancer treatment by simultaneously delivering functional miR-21 inhibitory oligonucleotide (miR-21i) and 5-fluorouracil (5-FU) using exosomes. This exosome-based 5-FU and miR-21i co-delivery system promoted cellular uptake and reduced the expression of miR-21 in 5-FU resistant HCT-1165FR cell lines expressing the Her2 gene. Downregulation of miR-21induced cell cycle arrest, lowered tumor growth, increased apoptosis, and rescued the expression of PTEN and hMSH2. Importantly, as compared to either mono-therapy, combined delivery of miR-21i and 5-FU effectively reversed drug resistance and increased the cytotoxicity in 5-FU-resistant colon cancer cells [130]. Exosomes containing oxaliplatin and PGM5-AS1 can also reverse drug resistance, offering another method for treating CRC [131]. Others developed tumor-derived exosomes for co-delivering aggregation-induced emission luminogens (AIEgens) and PPIs. This combined therapy was designed to promote AIEgens-based photodynamic therapy (PDT) via PPI-mediated inhibition of cell glutamine metabolism. Evaluation in a MGC803 gastric cancer subcutaneous model revealed that this exosome-based co-therapy can effectively prevent tumor growth and promote tumor immunogenic death [132].

## Exosomes as vaccines against cancer

Over the past half-century, therapeutic cancer vaccines (TCVs) have been investigated as a potential immunotherapeutic approach to treat cancer by stimulating CD8 + cytotoxic T cells to generate tumor-specific responses. TCVs have gained renewed enthusiasm due to their potential to improve the efficacy of checkpoint inhibition [133]. They target antigens specifically associated with malignant cells, resulting in fewer side effects and increased safety compared to existing cancer treatments. TCVs can be administered through various techniques using different antigens, adjuvants, and delivery vectors [134]. These techniques include peptide-, DNA/RNA-, and cell transfer-based cancer vaccines, each with its own advantages and disadvantages. Additionally, adoptive cell immunotherapies (ACTs) such as CAR-T and TIL have shown to be excellent antitumor therapies with strong and highly personalized immunogenic profiles. However, they are costly, time-consuming, and labor-intensive. [135–139]. Exosome vaccines have been introduced as a new platform for more efficient delivery of tumor-associated antigens, showing better efficacy than ACTs in eradicating tumors in a T cell-dependent and MHC-restricted manner. Exosomes combine processed peptides derived from antigenic material expressing surface MHC I/II and deliver functional peptide-MHC complexes to naive target cells, stimulating the expansion of peptide-specific clonal T cells and promoting tumor cytotoxicity through MHC-I and MHC-II antigen processing. Exosomal vaccines have shown superior efficacy compared to ACTs in eradicating tumors in a T cell-dependent and MHC-restricted manner [140, 141]. Understanding how exosomes activate antitumor immunity is crucial for advancing this promising immunotherapy. Exosomes deliver processed peptides expressing surface MHC I/II, stimulating target cells and promoting T cell activation [142, 143]. Several studies demonstrated that the best model of using exosomes is to load DCs with tumor antigens and subsequently extract the produced exosomes [144]. Loading DCs with tumor antigens and extracting the produced exosomes has been identified as an effective model. Exosomes stimulate the expansion of peptide-specific clonal T cells, promote CD8 + T cell maturation, and activate NF-κB in macrophages for tumor cytotoxicity through MHC-I. Additionally, antigen processing through MHC-II leads to more efficient activation of CTLs. Studies have demonstrated that different exosomes can effectively stimulate naive T cell proliferation and differentiation into cytotoxic T lymphocytes, leading to stronger killing activities against tumor cells [141, 144, 145]. A study examining DC-OVA-derived exosomes (EXODC) showed that EXODC can more effectively stimulate naive OVA-specific CD8 + T cell proliferation and differentiation into cytotoxic T lymphocytes in vivo as compared to EG7 tumor cell line-derived exosomes (TEXEG7); the stronger killing activities by EXODC against lung tumor cells were attributed to the expression of co-stimulatory molecules such as CD40 and CD80 [146]. Other work demonstrated that heat shock protein-70 (Hsp70)-enriched tumor exosomes increased the expression of MHCII and induced strong Th1 immune responses, eliminating CT26 (mouse colon carcinoma cells) cancer cells in allogeneic hosts [147]. These findings suggest that Hsp70 exosomes can be used as an innovative vaccination for the management of CRC [148, 149]. In a phase I clinical trial, ascites-derived exosomes (Aex) were used in combination with granulocyte–macrophage colony-stimulating factor (GM-CSF) in 40 patients with CRC. The results showed that treatment with the combined vaccine, but not Aex alone, triggered a specific anti-tumor CTL response, and was safe and well tolerated. Thus, immunotherapy using Aex in combination with GM-CSF could be considered as an effective vaccine in the treatment of patients with metastatic CRC [150]. The exosomes in addition of induction of strong immune responses, eliminate cancer cells, and modulate tumor progression. Furthermore, exosomes interact with immune cells to induce anti-metastatic effects and trigger specific anti-tumor CTL responses. In preclinical and clinical trials, exosomes have shown promise as cell-free anti-cancer vaccines. The potential of exosome-based cancer immunotherapy is highly promising and warrants further clinical validation [151].

## Conclusion

Recognition of the utilization of small extracellular vesicles for diagnostic and therapeutic purposes is fast growing. We will have an opportunity to advance the therapeutic use of exosomes as we learn more about their biogenesis and function. Furthermore, recent research has unequivocally established the value of exosomes as both natural drug delivery vehicles and biomarkers for disease diagnosis. Further advancement in the applicability of exosomes depends on the development of more precise and trustworthy separation techniques. In this regard, it is essential to combine basic science research with cutting-edge technology. For example, the use of exosomes as natural nanocarriers can have high potential in personalized treatments. To achieve this attractive goal, future research is required to fully understand the nature of exosomes in terms of their membrane composition and cargo. After identification of the desired properties of the exosome, it can be engineered in accordance with the therapeutic goals for treatment of various cancers, including gastrointestinal cancers. However, many challenges remain for the widespread use of exosomes in the clinic. Indeed, it is still incompletely understood how exosomes interact with the TME. In addition, it is unclear which cellular source of exosomes is safest and most efficient for the delivery of therapeutic agents, and techniques for targeting exosomes for clinical applications have not yet been optimally developed (there is a need for sensitive and accurate platforms). There are a multiple exosomal nucleic acids and proteins that can be used as biomarkers to detect cancers, selecting the best one is difficult. To facilitate the widespread clinical use of exosomes, it is necessary to be able to isolate and purify exosomes in a fast and cost-effective way. Furthermore, the clinical applicability of exosomes strongly relies on whether or not the many preclinical in vitro and in vivo findings can be successfully translated into clinical trial outcomes. Conceivably, in anticipation of clinical trial results and development of high-throughput technologies, exosomes have the potential to greatly improve and revolutionize cancer diagnosis as well as treatment strategies in the not-so-distant future.

## Data Availability

No datasets were generated or analysed during the current study.
